# The PI3K/Akt Pathway and Glucose Metabolism: A Dangerous Liaison in Cancer

**DOI:** 10.7150/ijbs.89942

**Published:** 2024-05-27

**Authors:** Fabrizio Fontana, Gaia Giannitti, Sara Marchesi, Patrizia Limonta

**Affiliations:** Department of Pharmacological and Biomolecular Sciences "Rodolfo Paoletti", Università degli Studi di Milano, Milan, Italy.

**Keywords:** PI3K, Akt, aerobic glycolysis, Warburg effect, cancer metabolism

## Abstract

Aberrant activation of the PI3K/Akt pathway commonly occurs in cancers and correlates with multiple aspects of malignant progression. In particular, recent evidence suggests that the PI3K/Akt signaling plays a fundamental role in promoting the so-called aerobic glycolysis or Warburg effect, by phosphorylating different nutrient transporters and metabolic enzymes, such as GLUT1, HK2, PFKB3/4 and PKM2, and by regulating various molecular networks and proteins, including mTORC1, GSK3, FOXO transcription factors, MYC and HIF-1α. This leads to a profound reprogramming of cancer metabolism, also impacting on pentose phosphate pathway, mitochondrial oxidative phosphorylation, de novo lipid synthesis and redox homeostasis and thereby allowing the fulfillment of both the catabolic and anabolic demands of tumor cells. The present review discusses the interactions between the PI3K/Akt cascade and its metabolic targets, focusing on their possible therapeutic implications.

## 1. Introduction

The phosphoinositide 3-kinase (PI3K)/Akt pathway is one of the most frequently deregulated molecular cascades in human malignancies 1. In physiological conditions, this signaling is triggered by growth factors, cytokines and hormones, and mediates different metabolic processes, particularly glycolysis, to support cell growth and survival. Oncogenic activation of the PI3K/Akt cascade leads to a wide metabolic reprogramming via upregulation of glucose transporters and glycolytic enzymes, also impacting on pentose phosphate pathway (PPP), mitochondrial oxidative phosphorylation, de novo lipid synthesis and redox homeostasis and thus fulfilling both the catabolic and anabolic needs of tumor cells 2. Based on these observations, a deeper understanding of the metabolic consequences of PI3K/Akt hyperactivation in cancers may open to the identification of novel therapeutic strategies. Herein, we provide an overview of the role of the PI3K/Akt signaling in the modulation of glucose metabolism and its associated biochemical pathways in malignant cells, with a focus on their potential as new pharmacological targets.

## 2. The PI3K/Akt pathway at a glance

**Figure 1:**
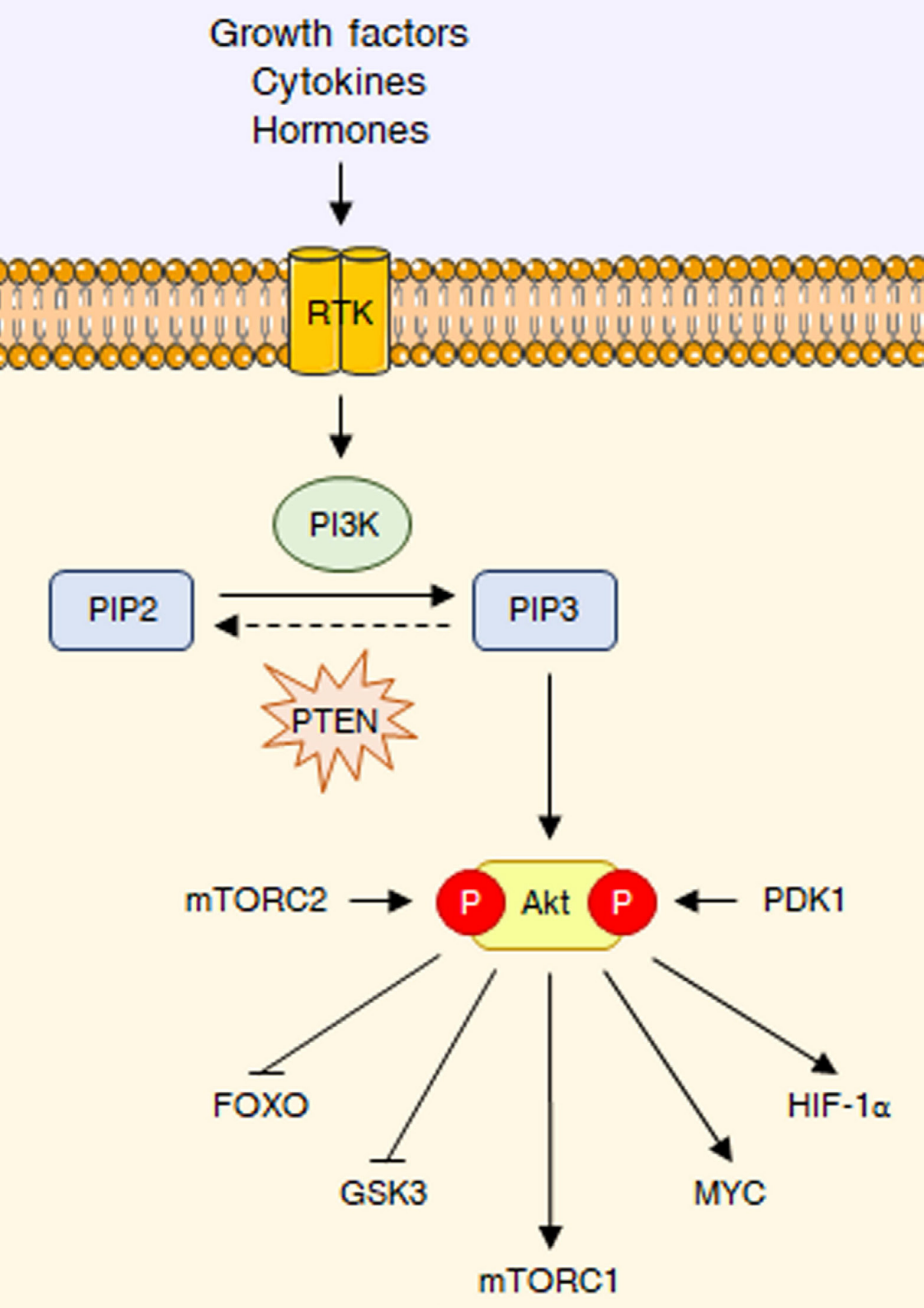
** The PI3K/Akt pathway at a glance.** RTK interaction with growth factors, cytokines or hormones leads to PI3K activation, which converts PIP2 into PIP3, that can be dephosphorylated back to PIP2 by PTEN. PIP3 acts as a second messenger to recruit Akt, which it is fully activated through phosphorylation at T308 and S473 by PDK1 and mTORC2, respectively. Akt reprograms glucose metabolism through different key downstream substrates, including FOXO transcription factors, GSK3, mTORC1, MYC and HIF-1α.

As previously mentioned, the PI3K/Akt signaling is deeply involved in the control of cell growth and proliferation 1. PI3Ks are plasma membrane-associated lipid kinases, consisting of two regulatory subunits (p85 and p55) and a catalytic subunit (p110) 3. Depending on their different structures and specific substrates, PI3Ks can be divided into three classes, I, II, and III: among these, PI3K IA represents the most commonly mutated protein in tumors 4. Under physiological conditions, PI3K is generally activated by a variety of extracellular stimuli, including insulin, growth factors and cytokines 3,5. Upon stimulation, PI3K mediates the phosphorylation of phosphatidylinositol 4,5-bisphosphate (PIP2) into phosphatidylinositol (3,4,5)-trisphosphate (PIP3), a second messenger able to bind to the pleckstrin-homology (PH) domains of a wide subset of kinases and promote their recruitment to the cell membrane; phosphatase and tensin homologue (PTEN) inhibits the pathway by dephosphorylating PIP3 to PIP2 3,5. One of the downstream targets of PIP3 is the serine-threonine kinase Akt (also called protein kinase B or PKB), which becomes fully active via subsequent phosphorylation at T308 by phosphoinositide-dependent protein kinase 1 (PDK1) and at S473 by mechanistic target of rapamycin complex 2 (mTORC2) 6. Akt governs glucose metabolism either directly, by phosphorylating multiple nutrient transporters and metabolic enzymes, or indirectly, by regulating different signaling networks and transcription factors, including mTORC1, glycogen synthase kinase 3 (GSK3), members of the forkhead box O (FOXO) protein family, MYC and hypoxia-inducible factor 1α (HIF-1α) 2. It should be noted that the genetic alterations responsible for the constitutive hyperactivation of the PI3K/Akt cascade are well-known cancer drivers. Common tumorigenic changes include: activating mutations in PIK3CA, the p110 catalytic subunit of PI3K1; deletion/loss of function in PTEN; amplification/activation of PI3K upstream regulators, particularly receptor tyrosine kinases (RTKs); amplification/gain of function in Akt 7. Overall, these modifications result in a plethora of biological effects, such as cell cycle dysregulation, apoptosis escape and epithelial-to-mesenchymal transition (EMT), ultimately culminating in cancer growth, metastasis and drug resistance 8. The main steps of the PI3K/Akt pathway are represented in **Fig. 1**.

## 3. The PI3K/Akt cascade and glucose metabolism in cancer

**Figure 2:**
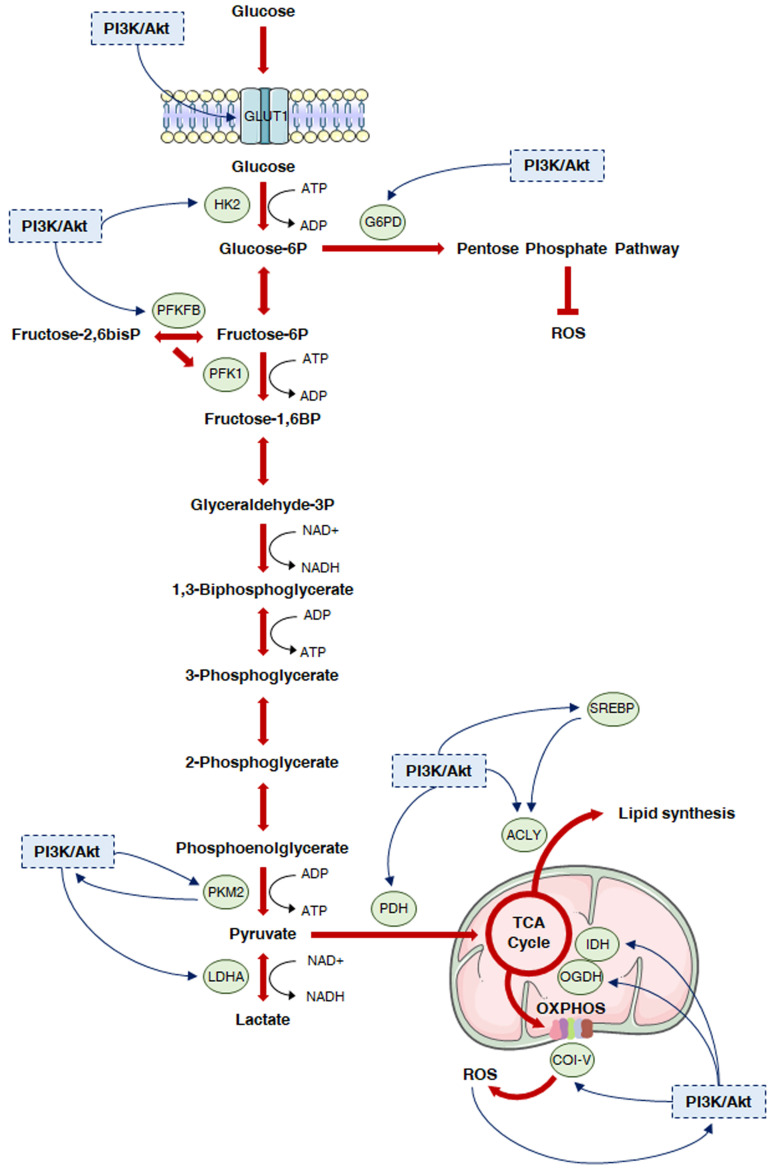
** The PI3K/Akt cascade and glucose metabolism in cancer.** The PI3K/Akt signaling is known to regulate glycolysis and different subsidiary metabolic pathways in cancer. In particular, it promotes plasma membrane localization of GLUT1, thus increasing glucose uptake. Then, it activates HK2 to facilitate the generation of glucose 6-phosphate, which can fuel both the PPP and the glycolytic flux. In this regard, the PI3K/Akt cascade can trigger nucleotide synthesis by inducing G6PD, while it can further enhance glycolysis by targeting PFKFB3 and 4, which generate the PKF1 activator fructose-2,6-bisphosphate, and PKM2, which is responsible for the production of pyruvate. Pyruvate is either converted into lactate by PI3K/Akt hyperactivation-induced LDHA or can enter the mitochondria; in these organelles, it undergoes a multistep oxidation via the TCA cycle and OXPHOS, that can be boosted by the PI3K/Akt pathway via upregulation of PDH, IDH and respiratory complexes I, III and IV. Moreover, the PI3K/Akt signaling directly stimulates lipid synthesis through direct or SREBP-dependent phosphorylation of ACLY, which generates acetyl-CoA from citrate. Finally, it controls redox homeostasis by replenishing NADPH cytosolic pool and modulating PDH, OGDH and OXPHOS complex I activity.

Deregulated glucose metabolism represents a distinctive trait of tumor cells. This peculiar phenotype, characterized by a high rate of glucose fermentation even in the presence of oxygen, was described by Otto Warburg in 1956 and is called aerobic glycolysis or Warburg effect 9,10. Besides producing ATP, the increase in the glycolytic flux allows glycolytic intermediates to supply subsidiary pathways implicated in the synthesis of various macromolecules, such as nucleotides, proteins and lipids, which are essential for cell growth and survival 9,10. Moreover, it favors the production of cofactors required for the maintenance of cellular redox state: enhanced glucose uptake allows higher generation of the reducing equivalent NADPH by the oxidative branch of the PPP, and lactic acid fermentation leads to the regeneration of the oxidizing equivalent NAD^+^ 11. Finally, it has been recently postulated that the Warburg effect might result from molecular crowding, a common limitation to overall metabolism in rapidly proliferating cells 12. Despite the enormous genetic heterogeneity of cancers, cell transformation appears to be based on the common induction of the PI3K/Akt signaling to promote the above metabolic rewiring (**Fig. 2**).

Glucose serves as a fundamental source of carbon and energy in all organisms. Its uptake into cells is facilitated by the family of glucose transporters (GLUTs), among which GLUT1 plays a pivotal role in tumorigenesis 13. Interestingly, a positive correlation between GLUT1, PI3K and p-Akt expression has been reported in several cancers, including endometrial and head and neck carcinoma 14-16. Mechanistically, it has been shown that Akt can promote the translocation of GLUT1 to the plasma membrane by phosphorylating and thus inhibiting thioredoxin-interacting protein (TXNIP), which mediates GLUT1 endocytosis and blocks glucose uptake 13,17. This results in an increased glycolytic flux, leading to enhanced tumor growth, metastasis and chemoresistance 18. Almost 40 compounds have been recognized as potential GLUT1 inhibitors. For example, many nutraceuticals, including a variety of alkaloids (i.e. matrine), flavonoids (i.e. apigenin, genistein and quercetin) and non-flavonoid phenolic molecules (i.e. resveratrol and curcumin), can suppress GLUT1 expression either directly or by affecting PI3K activity, while some synthetic drugs, such as BAY-876, STF-31 and WZB117, can interact with the transporter to inhibit glucose transfer 19,20. Unfortunately, none of these chemicals has been approved by Food and Drug Administration (FDA), due to low activity and poor specificity.

Glycolysis intensity is controlled by the activity of three physiologically irreversible enzymes: hexokinase (HK), phosphofructokinase-1 (PFK-1) and pyruvate kinase (PK). Regarding the first, four different forms exist 21. HK1, 2 and 3, which are characterized by a high affinity for glucose, consist of two similar 50-kDa N- and C-terminal lobes and can be inhibited by their product, glucose-6-phosphate; only C-terminal domain is functional in HK1 and 3, while both portions are endowed with catalytic activity in HK2. HK4 (also known as glucokinase) has only one 50-kDa lobe with reduced affinity for glucose. The expression of these isozymes varies across different tissues, with HK1 and less abundant HK3 being ubiquitously and stably expressed in all cells, HK2 being finely regulated by various hormonal or metabolic mechanisms in muscles and heart, and HK4 being mainly present in the liver, pancreas, small intestine and brain 21. Among them, HK2 is a specific downstream target of Akt in cancer 22-30. In particular, Akt has been observed to mediate the binding of HK2 to the voltage-dependent anion channel (VDAC) on the outer mitochondrial membrane (OMM), thereby enhancing its phosphorylating activity by allowing a direct exploitation of mitochondria-derived ATP 31-33. Of course, this is also followed by a reduced sensitivity to apoptosis, due to a limited opening of the mitochondrial permeability transition pore (mPTP) 31-33. The Akt/HK2 cascade is controlled by PH domain leucine-rich repeat protein phosphatase (PHLPP), which can dephosphorylate Akt and promote HK2 translocation from mitochondria to the cytosol 34. Remarkably, PHLPP is regulated by mTORC1 and in turn inhibits the mTORC1 substrate p70S6K 35, suggesting the existence of a complex regulatory system matching HK2 levels and subcellular localization with the metabolic status of the cell. A further characterization of HK2 functions in tumors has been recently provided by Ciscato et al., who have demonstrated that 80% of this protein lodges in mitochondria-associated membranes (MAMs), small subcellular regions connecting the OMM to the endoplasmic reticulum (ER); there, it can participate to the IP3R-GRP75-VDAC1-mediated control of Ca^2+^ homeostasis, contributing to the proliferation of cancer cells 36. Parallelly, Cheung and colleagues have reported the ability of HK2 to translocate to the mitochondrial surface under hypoxia and to bind to TIGAR, a fructose-2,6-bisphosphatase that inhibits glycolysis and promotes PPP; the obtained complex not only stimulates HK2 activity but also lowers mitochondrial ROS levels and protects against cell death 37. In this intricate scenario, it is worth underlying that HK2 is overexpressed in several human malignancies, such as liver, pancreatic, colon and ovarian carcinoma, and has been associated with tumor initiation and progression in many in vivo models 38-41. More importantly, it has been recently identified as a negative prognostic marker in glioblastoma multiforme and cervical and hepatocellular cancer, and its induction correlates with resistance to radiation, chemo- and targeted therapy 42-49. For these reasons, HK2 upregulation has been recently proposed as a metabolic vulnerability for the management of PTEN-deficient and Akt-overexpressing tumors, including prostate cancer 39,50-53. Indeed, HK2 can be successfully targeted by several drugs, such as the catalytic inhibitors lonidamine and 3-bromopyruvate (3-BrPyr) and the glucose-analogue 2-deoxyglucose (2-DG); however, the use of these compounds has been abandoned after the onset of severe side effects during clinical trials 54. On the other hand, different natural agents directed against HK2, including berberine, wogonin, epigallocatechin-3-gallate, β-escin and tocotrienols, have shown no toxicity when administered in vivo but little efficacy when given to patients 20. An alternative anti-HK2 strategy relies on the usage of specific peptides able to remove this specific enzyme from OMM without interfering with the activity of any other isoform. These chemicals contain specific sequences for plasma membrane crossing and can rapidly trigger tumor cell death via Ca^2+^ flux perturbation 32,36,55,56. Nonetheless, they lack selectivity for cancer cell entry, and their pharmacological utility still needs to be validated in pre-clinical and clinical settings.

As mentioned above, PFK-1 is another major rate-limiting enzyme of glycolysis, mediating the production of fructose-1,6-bisphosphate from fructose-6-phosphate. Its most potent activator is fructose-2,6-bisphosphate, a product of the reaction catalyzed by 6-phosphofructo-2-kinase/fructose-2,6-bisphosphatase (PFKFB). In humans, PFKFB is encoded by four genes, with PFKFB1 being found in the liver and skeletal muscle, PFKFB2 predominating in cardiac muscle, PFKFB3 being ubiquitously expressed and PFKFB4 occurring mainly in testes 57,58. Of note, PFKFB is both a substrate and positive effector of Akt pro-tumor activity. Indeed, isoenzymes 3 and 4 can be directly phosphorylated by this kinase 59; in turn, PFKB4 can promote Akt action by regulating the expression of histone acetyltransferase GCN5 or by interacting with isoprenylcystein carboxyl methyl transferase (ICMT), a posttranslational modifier of RAS 60,61. As discussed in the following paragraphs of the present article, PFKFB3 and PFKFB4 affect carcinogenesis in a multidirectional manner, participating in the control of glucose metabolism by enhancing not only glycolysis but also PPP 62. Unsurprisingly, high levels of these proteins have been found in breast, lung, colon, pancreatic, gastric, ovarian, thyroid and liver cancer, where they correlate with metastases to lymph nodes and advanced tumor staging 63-70. Thus, inhibitors of PFKFBs have been recently designed to improve therapeutic outcomes. In particular, new small molecules directed against PFKFB3, such as 3-(3-pyridinyl)-1-(4-pyridinyl)-2-propen-1-one (3PO), compound 26, PQP, KAN0438757 and PFK15, have shown promising anti-cancer effects in vitro and in vivo 59; more importantly, one of them, namely PFK158, has already been enrolled in a Phase I trial for the management of solid malignancies (clinicaltrials.gov #NCT02044861).

As one of the key glycolytic enzymes, PK acts on phosphoenolpyruvate to form pyruvate fueling the tricarboxylic acid (TCA) cycle in mitochondria 71. It has four different subtypes: PKL is mainly found in the liver, PKR is mainly expressed in red blood cells, PKM1 is distributed in myocardium, skeletal muscle and brain tissue, PKM2 is present in all proliferating cells, especially embryonic cells. PKL and PKR are encoded by the PKLR gene, while PKM1 and PKM2 are transcribed from the PKM gene via alternative splicing; in particular, PKM2 possesses the PKM2-specific exon 10 and lacks the PKM1-specific exon 9. This single exon difference results in important function distinctions: PKM1 constitutively oligomerizes to a highly active tetramer under physiological conditions, while PKM2 may be present as a tetramer or a less active dimer; this implies that the main biological function of PKM1 is the generation of ATP, whereas the tetramer-to-dimer transition of PKM2 supports the formation of glycolytic intermediates for biomass production. It should also be emphasized that several metabolites, such as fructose-1,6-bisphosphate, can act as allosteric activators of PKM2 but not of PKM1 71. Intriguingly, a switch from PKM1 to PKM2 has been detected in various cancers, and a reverse isoform shift from PKM2 to PKM1 has been found to inhibit aerobic glycolysis and reduce tumorigenesis in multiple xenograft models 71. In particular, recent findings point to an Akt-dependent pro-tumor modulation of PKM2 72-77, which has also emerged as an upstream regulator of the PI3K/Akt pathway itself 78-81; in particular, it seems to be able to activate mTORC1 by interacting with Akt1 substrate 1 (Akt1S1) 82. In this context, aberrant PKM2 expression has been widely associated with poor prognosis in patients with solid tumors of the digestive system, such as esophageal, gastric, colorectal and liver carcinoma 83-85. There are different small molecules able to reduce tumor growth by targeting PKM2 20,86-88. These drugs, particularly shikonin and its analogs, bind to the allosteric site of the enzyme, thereby impairing glucose metabolism 20,86-91; nevertheless, they have only been tested in vitro.

Lactate production largely contributes to malignant progression, not only by replenishing NAD^+^ for glycolysis itself but also by lowering extracellular pH for invasion and triggering angiogenesis and immune escape 92. Lactate dehydrogenase A (LDHA) is the main effector of glucose fermentation, converting pyruvate into lactic acid 92. Increased levels of this enzyme have been reported in numerous malignancies, including pancreatic, nasopharyngeal, gastric, bladder and endometrial cancer, and are commonly linked to PI3K/Akt hyperactivation 92-101. Considering this, LDHA has been regarded as an intriguing target for tumor prevention and therapy, with pyruvate- and NADH-competitive inhibitors (i.e. oxamate, gossypol, FX11, quinoline 3‐sulfonamides and N‐hydroxyindoles) and free enzyme-binding molecules (i.e. galloflavin) demonstrating great efficacy in a variety of cellular and xenograft cancer models 92. Yet, according to recent literature, LDH inhibition can disrupt the metabolism of healthy T cells, causing severe immunosuppression 102; to overcome this issue, lactate oxidase/catalase-displaying nanoparticles have been proposed as useful tools to consume lactate in the tumor microenvironment and effectively suppress cancer growth 103,104.

Besides directly controlling the activity of glucose transporters and glycolytic enzymes, the PI3K/Akt signaling can drive the Warburg effect by modulating the expression of MYC. This transcription factor is one of the most encountered oncogenes in tumors, where it favors aerobic glycolysis by upregulating GLUT1 and most metabolic enzymes, including HK2, PFK-1, enolase 1 (ENO1), pyruvate dehydrogenase kinase 1 (PDK1), PKM2 and LDHA 105. The PI3K/Akt pathway promotes MYC induction via various molecular mechanisms 105. For instance, mTORC1 increases MYC translation 106,107, while Akt stabilizes the protein via inactivation of the proteasomal degradation inducer GSK3 108-110. Moreover, the PI3K/Akt cascade can inhibit the suppressive effects of FOXO transcription factors on MYC-mediated increase in glycolytic flux 111. However, it should be emphasized that MYC expression can be finely controlled by a wide range of oncoproteins, including mitogen-activated protein kinases (MAPKs) 112-114. For this reason, the MYC-related reprogramming of glucose metabolism cannot be exclusively reconducted to the action of Akt.

A distinctive hallmark of several malignancies is that they develop in a hypoxic environment 115. The metabolic adaptation to hypoxia is orchestrated by HIF-1, a heterodimeric transcription factor composed of two subunits, α and β. In the presence of oxygen, HIF-1α is hydroxylated at pro402 and pro564 by prolyl hydroxylases, resulting in its ubiquitination and proteasomal degradation. Under hypoxic conditions, prolyl hydroxylases are inhibited, and HIF-1α can thus translocate to the nucleus and dimerize with its partner HIF-1β. The HIF-1 dimer then binds to the hypoxia response element site on DNA, inducing the expression of a variety of genes implicated in the hypoxic response; among them, there are numerous enzymes participating in the control of the glycolytic flux, such as HK2, PFK-1, fructose-bisphosphate aldolase A (ALDOA), ENO1, PKM2 and LDHA 116. Intriguingly, many cancers exhibit constitutive activation of HIF-1α even in normoxic conditions, and Akt is known to participate in this process via mTORC1 upregulation 117-122. In this context, HIF1α stabilization seems to directly contribute to tumor growth, presumably endowing the tumor mass with the metabolic flexibility necessary to adapt to oxygen fluctuations.

Emerging evidence suggests that MYC and HIF also cooperate to promote the metabolic reprogramming observed in Akt-overexpressing malignancies 123. Under hypoxia, MYC is inhibited by HIF-1α through disruption of the MYC/MAX complex, resulting in adaptive cellular changes that favor survival in low-oxygen conditions. On the other hand, when MYC is overexpressed, it reduces HIF-1α degradation and increases its activity at the chromatin levels, directly participating in the boost in the glycolytic flux 123. In particular, it has been recently reported that mTOR-related upregulation of PKM2 in mouse kidney tumors results from HIF-1α-mediated transcription activation and c-Myc-heterogeneous nuclear ribonucleoprotein (hnRNP)-dependent regulation of PKM2 gene splicing 124. Given the importance of the MYC-HIF interplay in cancer cells, it would be critical to further understand how these proteins precisely interact with each other to jointly regulate the expression of metabolic genes.

Cancer onset and evolution are not only influenced by genetic/epigenetic changes in malignant cells but also by the rearrangement of the components of the tumor microenvironment through a bidirectional and dynamic crosstalk 125. Among the different cell types that are found in tumor stroma, adipocytes have emerged as critical regulators of cancer metabolism 126. In particular, periprostatic adipose tissue has been found to induce tumor switch towards the Warburg phenotype through Akt/HIF-1α activation 127. Likewise, bone marrow adipose cells have been shown to fuel the glycolytic pathway of metastatic prostate carcinoma by promoting Akt phosphorylation 128. Remarkably, these metabolic alterations seem to be mediated by both soluble and insoluble extracellular factors.

## 4. The PI3K/Akt cascade and other glycolysis-associated metabolic processes

The above evidence emphasizes the crucial role played by the PI3K/Akt signaling in the induction of aerobic glycolysis in cancer. In addition to accelerating ATP production, this phenotypic switch improves the metabolic flux into various glucose-dependent pathways that are responsible for the synthesis of cellular macromolecules 129. As discussed in the following paragraphs of this article, these biosynthetic mechanisms are also under control of the PI3K/Akt cascade (**Fig. 2**).

### 4.1 Pentose phosphate pathway

Nucleotides serve as monomeric units of DNA and RNA 130. In tumors, de novo synthesis of these molecules is commonly exacerbated to sustain unlimited cell growth and proliferation 131. This process is supported by different metabolic mechanisms, including the glucose-dependent PPP. The PPP consists of two reaction sequences, often referred to as the oxidative and non-oxidative branches. In the oxidative arm, glucose 6-phosphate (G6P) is converted to ribulose 5-phosphate (Ru5P) and CO_2_, with the formation of two molecules of NADPH essential for both anabolic reactions and ROS scavenging; in the non-oxidative phase, three molecules of Ru5P are converted to two molecules of fructose 6-phosphate (F6P) and one molecule of glyceraldehyde 3-phosphate (GAP) 132. Glucose-6-phosphate dehydrogenase (G6PD) is the rate-controlling enzyme of the PPP, promoting the oxidation of G6P to 6-phosphoglucono-δ-lactone. By boosting the glycolytic flux, the PI3K/Akt signaling not only provides multiple co-factors for nucleotide generation, such as ATP, NADH and NADPH, but also increases the production of G6P 133; on the other hand, it specifically promotes the induction of G6PD expression 122. Of note, Akt can also upregulate the PPP by controlling the activity of other enzymes, either directly - as in the case of transketolase (TKT) 134,135 - or indirectly - by stimulating the MYC-related transcription and translation of phosphoribosyl pyrophosphate (PRPP) synthase 2 (PRPS2) 136,137. Finally, it should be emphasized that Akt-dependent regulation of PFKFB3 and PFKFB4 levels might considerably impact on the crosstalk between glycolysis and PPP 138. Indeed, it has been shown that inhibition of PFKFB3 reroutes glucose metabolism from glycolytic flux to PP synthesis; conversely, silencing of PFKFB4 causes increases in fructose-2,6-bisphosphate concentrations, which in turn divert more glucose into glycolysis at the expense of PPP and NADPH production. The differences in the function of PFKFB3 and PFKFB4 apparently allow malignant cells to exploit these isozymes to allocate glucose to either the glycolytic process or the PPP, finely regulating the balance between cellular bioenergetics and redox homeostasis 138.

### 4.2 Oxidative phosphorylation

Contrary to Warburg's original hypothesis that aerobic glycolysis is a consequence of impaired mitochondria, recent investigations have clearly established that mitochondrial function is still intact in many cancers 62,139-142. In particular, it has been largely demonstrated that oxidative phosphorylation (OXPHOS) substantially contributes to ATP production and redox balance in tumor cells. Despite being at a preliminary stage, recent studies indicate that Akt exerts multiple activities in the control of mitochondrial metabolism, affecting both the TCA cycle and OXPHOS. For example, this oncoprotein has been shown to phosphorylate the catalytic subunit of pyruvate dehydrogenase (PDH), namely PDH-E1α, with great impact on acetyl-CoA generation and overall energy homeostasis 143. Furthermore, it has been demonstrated to regulate the sterol regulatory element-binding protein (SREBP)-mediated transcription of all three isocitrate dehydrogenase (IDH) isoforms (1, 2 and 3), thus boosting the TCA cycle and linking mitochondrial respiration with lipid synthesis 144. Finally, improved ATP production as a consequence of active Akt has been associated to mTORC1- and 4E-BP1-related modulation of the respiratory complexes I, III and IV 145. In the light of this, many efforts are currently being made to develop safe and effective anti-cancer approaches directed against mitochondria, as extensively reviewed in 146,147.

As reported above, the Warburg Effect only partially describes the complexity of cancer metabolism. Recent studies have shown that aerobic glycolysis and OXPHOS are not mutually exclusive, with tumor cells displaying flexible metabolic phenotypes that shift in relation to the specific conditions within the microenvironment 148. In this context, a newer theory, known as the “reverse Warburg effect”, defines a two-compartment model where cancer-associated fibroblasts (CAFs) are forced by malignant cells to undergo an Akt-dependent increase in the glycolytic flux and then transfer the lactate back to the tumor to fuel mitochondrial OXPHOS 149,150. These dual interactions allow cancers to quickly respond to changes in nutrient availability to maximize cell proliferation and survival and are primarily mediated by monocarboxylate transporters (MCT): MCT4 promotes the release of lactate from CAFs and is upregulated by HIF-1α and NF-κB; MCT1 is predominantly involved in the uptake of this catabolite by cancer cells and is induced by MYC and TIGAR 149,150. Of course, there are important clinical implications associated with the expanding knowledge of the tumor metabolic heterogeneity responsible for the reverse Warburg effect. Indeed, both cancer and stroma metabolic parameters might be incorporated into more reliable prognostic models, improving the prediction of disease behavior and outcomes. On the other hand, tumor-stroma metabolic coupling may represent a novel target for anti-cancer strategies.

### 4.3 De novo lipid synthesis

Whereas non-malignant tissues depend on extracellular fatty acids for their growth and survival, cancer cells increase de novo lipid biosynthesis to rapidly proliferate 144. Both glycerol-3P from glycolysis and citrate from mitochondrial respiration are essential building blocks for lipid synthesis, evidencing the ability of the PI3K/Akt signaling to indirectly coordinate lipid metabolism 151,152. On the other hand, Akt has been found to specifically modulate the expression of key enzymes controlling both fatty acid and cholesterol production.

ATP citrate lyase (ACLY) acts as a link between carbohydrate metabolism and fatty acid biosynthesis, by converting citrate into acetyl-CoA. The PI3K/Akt cascade can directly phosphorylate this enzyme and increase its activity, thus favoring tumor growth and affecting histone acetylation 153,154. ACLY overexpression is commonly observed in a variety of human malignancies, and its targeting reduces cancer cell proliferation both in vitro and in vivo 155; based on this evidence, ACLY inhibitors, originally designed for metabolic disorders, are now being tested as potential anti-cancer drugs 156.

Akt can also regulate de novo lipid synthesis by inducing SREBPs 157. When inactive, these transcription factors are located within the endoplasmic reticulum; their activation requires the cleavage into a N-terminal domain which is translocated to the nucleus. Once activated, they bind to specific sterol regulatory element DNA sequences, thereby upregulating the synthesis of several enzymes implicated in fatty acid generation, including ACLY; after that, SREBP mature forms are targeted for ubiquitin-dependent degradation by GSK3-mediated phosphorylation 158. The PI3K/Akt pathway promotes the above processing via mTORC1 induction and stimulates protein stabilization through GSK3 inhibition 159,160. Recently, MYC has also been found to contribute to SREBP-mediated lipogenesis in tumors 161. Of note, SREBP levels are known to be elevated in various malignancies, thus representing intriguing targets for cancer management 162.

### 4.4 Redox homeostasis

Reactive oxygen species (ROS) are biological byproducts of multiple biochemical processes, including glucose metabolism. Interestingly, moderate ROS generation has been shown to support cancer cell proliferation and survival, whereas higher levels of these molecules are detrimental to intracellular components 163. In this context, the PI3K/Akt pathway stimulates both ROS-producing and -scavenging mechanisms that vary between malignant settings. For instance, ROS have been reported to promote tumor growth by activating the PI3K/Akt signaling via PTEN inhibition or direct upregulation of Akt protein 164. Parallelly, the PI3K/Akt cascade has been found to boost the generation of superoxide and H_2_O_2_ by abolishing the negative regulation imposed by mitochondrial GSK-3β on PDH and OXPHOS complex I 164. In addition, oncogenic PIK3CA is known to confer a peculiar metabolic state to cancer cells, characterized by the overexpression of oxoglutarate dehydrogenase (OGDH): this enzyme is not only an important ROS source but also plays a key role in fueling the malate-aspartate shuttle, which is fundamental for the cytoplasmic NAD^+^ regeneration that supports the rapid glycolytic flux occurring in Akt-overexpressing tumors 165. On the other hand, Akt is able to increase the cellular reducing power available for antioxidant responses. As illustrated above, it can replenish the cytosolic pool of NADPH by boosting the oxidative branch of PPP. Moreover, it promotes the activity of NAD kinase (NADK), which catalyzes the phosphorylation of NAD^+^ into NADP^+^, the rate-limiting substrate for NADPH-producing enzymes 166. Finally, it contributes to ROS neutralization through stabilization and sustained activation of NRF2 (nuclear factor erythroid 2-related factor 2), a transcription factor that controls the expression of numerous antioxidant genes, including those of the glutathione and thioredoxin systems 167. Overall, this evidence points to the attractive therapeutic possibility of altering redox homeostasis in cancers exhibiting PI3K/Akt hyperactivation, by either reducing intracellular ROS content or promoting an excessive ROS production 164,168. In this regard, the apparent cytotoxicity of ROS increase has been recently exploited in the management of PI3K inhibitor-resistant malignancies. Specifically, resistant cells cultured in the absence of PI3K inhibitors (mimicking drug holidays conditions) have been found to develop a proliferative defect due to an mTORC1-dependent accumulation of ROS 169. Remarkably, these anti-proliferative effects can be counteracted by treatment with ROS scavengers, such as N-acetylcysteine (NAC), therefore highlighting the redox vulnerabilities of the drug-insensitive cell subpopulations emerging through therapies 169. Of course, despite these promising data, it is important to underline that the pharmacological exploitation of ROS-associated mechanisms in oncology is complex. Indeed, there is still significant debate about the possible systemic consequences of ROS level elevation in cancer patients as well as regarding the actual benefits of antioxidants in cancer therapy 170. Hence, it is fundamental to deeply characterize tumors at both the genetic and metabolic levels to establish whether targeting their redox homeostasis might represent an appropriate approach for their elimination.

## 5. Clinical implications

Due to its crucial role as an oncodriver, the PI3K/Akt pathway remains a prime candidate for therapeutic intervention. However, only five PI3K inhibitors (copanlisib, idelalisib, umbralisib, duvelisib and alpelisib) have been approved by FDA so far, while Akt inhibitors, such as MK-2206, AZD5363, GDC-0068 and perifosine, are still being tested in phase I and II clinical trials 171,172. In particular, most drugs suffer from many adverse effects (i.e. hyperglycemia and consequent hyperinsulinemia) as well as from poor solubility and permeability. Moreover, they have demonstrated limited therapeutic benefit as single agents. Therefore, designing new molecules based on different binding sites could help increase selectivity and reduce toxicity. On the other hand, focusing on other pathways by exploiting targeted and endocrine therapies as a combinatorial approach could enhance the efficacy of available agents 171,172. As discussed above, pharmacological targeting of specific metabolic transporters and enzymes downstream of the PI3K/Akt signaling might offer interesting alternative anti-cancer strategies to PI3K and Akt inhibitors (**Table 1**).

## 6. Conclusions and future perspectives

A growing body of evidence suggests that aberrant activation of the PI3K/Akt cascade drives tumor initiation and progression via a profound rewiring of glucose metabolism. In this context, future studies should be focused on a deeper characterization of the critical regulatory nodes connecting this signaling to the glycolytic flux in different malignancies, to identify new metabolic dependencies and vulnerabilities. On the other side, the interactions between the PI3K/Akt pathway and other glycolysis-related metabolic networks should be investigated, to develop a broader view of the tumorigenic implications of Akt constitutive phosphorylation. Indeed, metabolic enzymes are inherently druggable and could represent fundamental therapeutic hotspots, due to their role as essential modulators of cancer cell proliferation and survival.

Finally, it should be taken in consideration that the specific features of the stroma surrounding both the primary and metastatic tumor as well as the nutritional status of the host differentially influence the impact of PI3K/Akt signaling on cancer metabolism, further supporting the usage of combinatorial anti-metabolic strategies in tumor treatment.

## Figures and Tables

**Table 1:** Main drugs targeting glycolysis in cancer.

Drug	Target	Development phase	Main results obtained so far	Ref.
Matrine, apigenin, genistein, quercetin, resveratrol and curcumin	GLUT-1 downregulation	In vitro and vivo studies, Phase I and II trials	High activity in vitro, no toxicity but modest efficacy in vivo and in patients	19,20
				
BAY-876, STF-31 and WZB117	Inhibition of GLUT-1 function	In vitro and in vivo studies	High activity in vitro, no toxicity but modest efficacy in vivo	19,20
3-bromopyruvate and lonidamine	Catalytic inhibition of HK2	Phase I, II and III trials	High clinical efficacy but severe toxicity	54
				
2-deoxyglucose (2-DG)	Allosteric and competitive inhibition of HK2	Phase I, II and III trials	High clinical efficacy but severe toxicity	54
Wogonin, epigallocatechin-3-gallate, β-escin and tocotrienols	HK2 downregulation	In vitro and in vivo studies, Phase I and II trials	High activity in vitro, no toxicity but modest efficacy in vivo and in patients	20
				
Specific anti-HK2 peptides	Selective removal of HK2 from outer mitochondrial membrane	In vitro studies	High activity	32,36,55,56
				
3PO, compound 26, PQP, KAN0438757, PFK15 and PFK158	Inhibition of PFKB3 function	In vitro and in vivo studies, Phase I trials	High activity in vitro, high efficacy and no toxicity in vivo and in patients	59
Shikonin and its analogs	Allosteric inhibition of PKM2	In vitro studies	High activity	20,86-91
Oxamate	Pyruvate‐competitive inhibition of LDHA	In vitro studies	High activity	92
Gossypol, FX11 and quinoline 3‐sulfonamides	NADH‐competitive inhibition of LDHA	In vitro and in vivo studies	High in vitro activity and in vivo efficacy	92
N‐hydroxyindoles	Pyruvate‐ and NADH-competitive inhibition of LDHA	In vitro studies	High activity	92
Galloflavin	Inhibition of LDHA function	In vitro studies	High activity	92
